# Plastic Surgery—Myths and Realities in Developing Countries: Experience from Eastern Nepal

**DOI:** 10.1155/2011/870902

**Published:** 2011-12-08

**Authors:** Brijesh Mishra, Robin Koirala, Nalini Tripathi, Kajan Raj Shrestha, Buddhinath Adhikary, Surendra Shah

**Affiliations:** ^1^Department of Plastic Surgery, C.S.M. Medical University, Lucknow 226003, India; ^2^Department of Surgery, B.P. Koirala Institute of Health Sciences (BPKIHS), P.O. Box 7053, Dharan, Nepal; ^3^Department of Dentistry, Hind Institute of Medical Sciences, Barabanki 225001, India

## Abstract

B.P. Koirala Institute of Health Sciences, Dharan, Nepal, is the only tertiary care referral centre in the eastern region of Nepal. This paper discusses the author's experience of starting a plastic surgery unit in eastern Nepal regarding need and present status of plastic surgery care in Nepal. *Methods*. We analyzed the data of patients treated in Plastic surgery unit from July 2007 to February 2009. We did evaluation regarding type of patients, procedures, and their outcome. We also evaluated the limitations and their possible solutions to overcome the barriers to establish effective plastic surgical centers in developing countries. *Results*. Plastic surgery services were started as a unit in general surgery by single plastic surgeon and one general surgery resident on rotation. Total 848 patients were treated for different plastic-surgery-related conditions, which included 307 acute burn patients 541 general plastic surgery patients. Trauma constituted the major bulk 22%, followed by tumors 20%, while aesthetic surgery operations were only 10.1%. *Conclusions*. In developing countries, aesthetic procedures constitute very small part of plastic surgery interventions and plastic surgery units are primarily required for reconstructive needs for optimum management of patients.

## 1. Introduction

Plastic surgery is a special branch of medicine that deals with correction of head-to-toe disfigurement and other anomalies in the physical form that are either congenital or acquired. In developing countries, plastic surgery is usually considered a nonessential super specialty service as it is commonly correlated with cosmetic surgery and hence, it is one of the last departments to be started in a government hospital. Advancements in technology have broadened the scope of plastic surgery and today it is performed for a variety of reconstructive and aesthetic purposes. 

This paper briefly discusses the author's experience of plastic surgery services in the eastern region of Nepal; it discusses the demand of masses, limitations of infrastructure, and future perspectives in development of plastic surgery in eastern Nepal.

## 2. Material and Methods

B.P. Koirala Institute of Health Sciences receives patients from different parts of eastern Nepal and neighbouring Indian states, namely, Bihar and west Bengal. This study would highlight the relevant observations, limitations, and suggestions for better fulfilment of the objectives of a plastic surgical centre for eastern Nepal.

 Plastic surgery services were provided by single plastic surgeon and one resident as a specialty unit in the Department of Surgery. We collected data for all admitted patients and day care procedures/minor outpatient procedures, by reviewing different records. It does not include data for all out patient consultations as many of them were referred from other outpatient departments.

 We analyzed the data from July 2007 to February 2009 regarding type of patients, patient care, different procedures, and their outcome.

## 3. Results

From July 2007 to February 2009, 848 patients were treated for different plastic-surgery-related conditions. Out of these 848 patients, 307 were acute burn patients (36.2%) and 541 general plastic surgery patients (63.7%) (Table 1). 

Out of these 541 patients, 296 required operative intervention and 245 patients were managed conservatively for conditions like keloids, hypertrophic scar, pigmented lesions, nonhealing ulcers, and wounds. These patients were managed by local steroid injections, sclerotherapy, minor debridements, splintage, and so forth (Table 1). Out of these 541 patients, 195 were inpatients (36%) and the rest were treated as outpatients.

Out of 296 patients operated, only 30 cases were operated in emergency which was mainly limb/life-saving surgeries for example, reimplantations, revascularizations along with some flap cover and emergency fasciotomy or amputations (Figure 3).

Majority of patients were operated in Department of General Surgery (88.8%), followed by Orthopaedics, ENT, Dentistry and Ophthalmic Departments (Table 2). Out of 296 operations; 174 were major surgeries and 101 minor surgeries Table 3.

Trauma constituted the major bulk of plastic surgery intervention (22%) followed by tumors (20%), burn contractures (15.2%), and congenital deformities (14.1%), while aesthetic surgery operations were only 10.1%. Various inflammatory and chronic infectious conditions were kept in miscellaneous category which accounted for the rest of 18.6% (Table 3). 

Microvascular surgery was done for 21 patients, which included 6 free flaps, 4 reimplantations, and 11 revascularizations. Longest operative time was 10 hours for reimplantation of multiple fingers, while average time for hand reimplant and free flap surgery was 6–8 hours.

**Figure 1:**
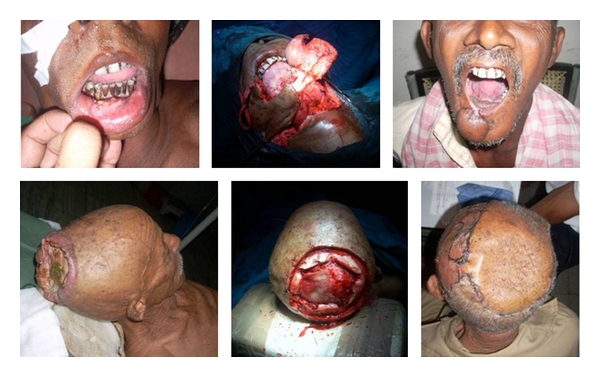


Oncological reconstructions were done for 59 patients. This included 5 free micro vascular flaps, 43 pedicled flaps, and skin grafting or direct closure for 11 patients. Majority of them were head and neck cancers followed by extremities and breast. Excision and reconstructions were done by Plastic Surgery Unit along with help of General Surgery and Otorhinolaryngology Department in absence of specialist cancer surgeons (Table 4, Figure 1).

20 cases of arteriovenous and lymphatic malformations required operative excision and closure with or without local flaps. Other congenital anomalies included clefts [1] and hypospadias [2] hands and foot anomalies (Figure 4).

**Figure 2:**
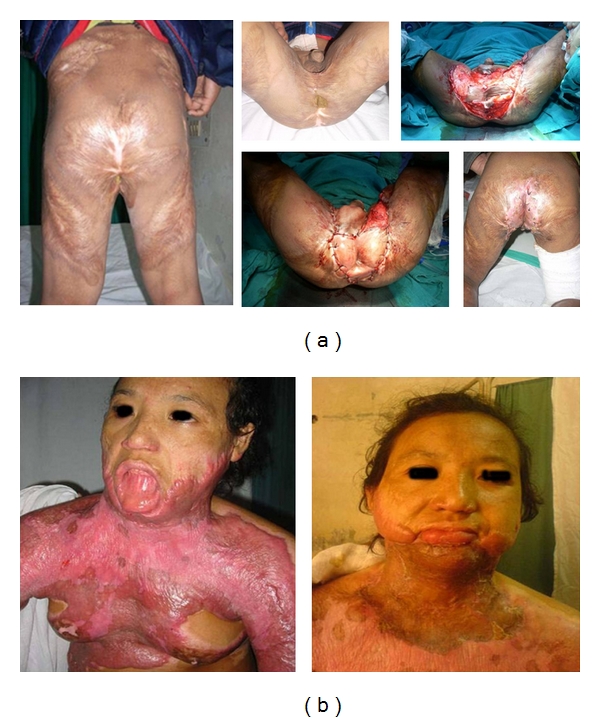


**Figure 3:**
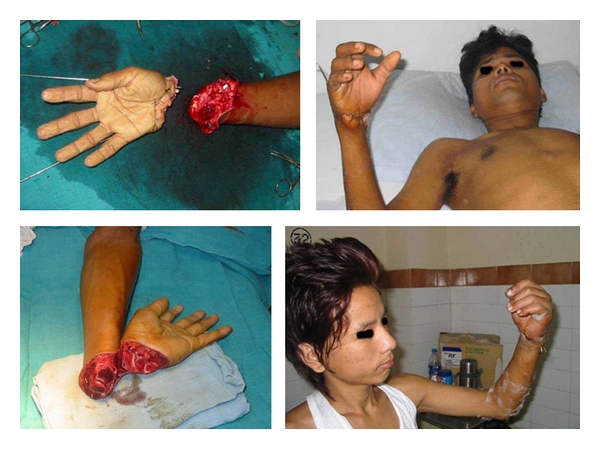


**Figure 4:**
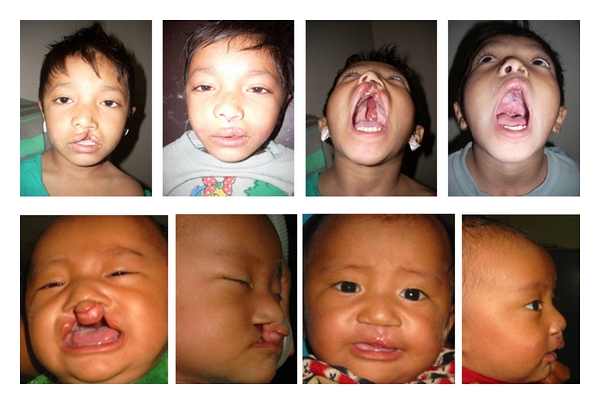


45 cases of postburn deformities required surgical correction for contractures of neck, axilla, elbow, hands, knee, and perineum. These included neglected long-standing contractures with severe morbidities (Figures 2(a) and 2(b)). 

Two patients, one patient of craniocerebral trauma with extrusion of brain matter and one patient of squamous cell carcinoma scalp with involvement of parietal bones required craniotomy and reconstruction in absence of neurosurgeon (Figure 1).

Three patients of panfacial fractures were treated in absence of oral maxillofacial surgeon. 

Three patients required excision of femoral artery pseudoaneurysm in drug abusers and one patient was operated for postgunshot common carotid artery pseudoaneurysm in absence of vascular surgeon. 

The majority of the patients improved after treatment. There was no mortality in operative patients. There was no significant morbidity except in 4 patients who had total flap loss. Two of them were free microvascular flaps and two pedicled flaps (Table 5). 

Out of 307 burn patients' majority required admission (88%). Superficial burns of less than 25% body surface area were treated as outpatients. Mortality rate for acute burn patient was 15.1% for total duration. There was yearwise reduction in mortality after a functioning plastic surgery unit (12.32% in year 2008 in comparison to 17.92% in the year 2007) (Table 5). 

Hospital stay of admitted patients was variable and many patients stayed for long durations as they belonged to remote hilly areas with difficult access to primary health centres and need for second surgery for detachment/adjustments.

Plastic surgery unit had 6 beds for burns and 6 beds for plastic surgery patients in the General Surgery ward. The hospital had 8 bedded ICU which supported major Head and neck onco reconstructions, but no burn patients could avail ICU services because of lack of separate beds and fear of infection. 

There were 2 outpatient days and 1 full operating day per week allocated to the specialty. Of 10 residents in general surgery, only one resident each month was assigned to plastic surgery on rotation basis.

Operation theatre was well-equipped for general surgery cases but there were no specific plastic surgery equipments for palatoplasty, microsurgery, or craniofacial surgery, and so forth. 

The author used magnifying loupes of 4.5x for all cases requiring microvascular surgery. It was a good enough magnification for reimplantations at wrist level, for most of the free flaps like radial artery flap, free fibula flap, and anterolateral thigh flap. The author encountered a problem of magnification for smaller vessels like finger replants and for free gracilis flap which resulted in failures. Handling of 10–0 nylon suture was difficult under loupe magnification. Monitoring was purely clinical by temperature and pin prick method done by the author and his residents. Two microvascular free flaps needed reexplorations and both could not be salvaged. 

The anaesthesia support was good with modernized trolleys and monitors to handle paediatric patients and prolong surgeries. 

The hospital had good radiology services with MRI and CT scans.

## 4. Discussion

Plastic surgery is derived from a Greek word “Plastikos” which means “to mould.” Earliest known procedures were described in an Indian publication, The Samhita, about 600 B. C. [3].

Plastic surgery as specialty started taking shape in the period after World War I, when war victims with complex problems required reconstructive surgeries. For the most part in the world, World War II brought a period of refinement in plastic surgery. 

Aesthetic surgery became popular in western countries in last few decades with changing socioeconomic conditions. Awareness for reconstructive plastic surgery services is generally lacking in public and even among doctors because of media hype for aesthetic surgery.

Plastic surgery services are in infancy in Nepal. There are few local plastic surgeons in capital city, and the rest of Nepal has some occasional visiting plastic surgeons for short term [4, 5]. There are very few articles in the literature regarding plastic surgery procedures in Nepal [4–8].

Eastern Nepal has one tertiary care centre and has good infrastructure for basic specialties. There is shortage of super specialists in all the fields and plastic surgery is no exception. Patients have to go to either India or Kathmandu for their plastic surgery needs. 

In Nepal, the total government health expenditure per capita is US $52, and the total health expenditure is only 5.1% of gross domestic product which is comparable to other south Asian countries (India 86$/3.6%, Pakistan 47$/2.0%, and Bangladesh 37$/3.2%) but far less than developed world (USA 6719$/15.3%) [1]. 

Almost no health insurance exists in Nepal. Patients and their families are expected to bear the total cost of medical treatment, which often means that they must sell land or borrow money. Added to these costs are other medical supplies required by the patient.

This cost of treatment is further compounded for many Nepalese patients, who must travel from their rural communities to the hospital. Lack of accessibility of medical care is a common reason for delayed presentation to tertiary care centres [5].

## 5. Existing Problems

The following parameters are identified as constraints in the care and training of plastic surgery in Nepal:

- political instability and lack of consciences on health issues to improve facilities,
-  inadequate organizational structure and training for plastic surgery,
-  inadequate lines of responsibility and accountability,
-  inadequate clinical audit systems and record keeping,
-  lack of proper guidelines by the hospital and difficulty in applying approved guidelines due to limited resources.

The root cause of most constraints is ongoing political instability which is reflecting on various levels of administration. It is expected to improve after formulation of proper constitution which is under process even at present. 

## 6. Strategies for Improvement

The most essential step is to initiate a proper training program in plastic surgery to produce dedicated plastic surgeons [2]. Plastic surgery training program has been started at T.U. Teaching Hospital, Kathmandu which will ensure continuous production of trained plastic surgeons. 

All tertiary care centres should have facilities for plastic surgery patients and for Burns. The institute should have guidelines for development of multispecialty team approach to develop tertiary care centres in real sense with development of all specialities. 

There are many nongovernmental organizations working in Nepal to help patients of congenital deformity and burns for example, Smile Train, Interplast, and so forth. Institutions should collaborate with these organizations for development of better infrastructure and services for patients and better training for medical staff. The author tried to get collaboration of Smile train for cleft patients and the centre was inspected and accredited by Smile train, but the programme could not be started as the author left the place. 

Our observation shows that the role of plastic surgeon becomes more vital in centres where other specialty services are lacking, as plastic surgeons can handle variety of situations because of their training [2]:

- treatment of vascular emergency in absence of vascular surgeons,
- treatment of maxillofacial injuries in absence of oral maxillofacial surgeons,
- treatment of complex head injury cases requiring flap covers along with basic support from general surgery,
- treatment of cutaneous malignancy and head neck cancers in absence of onco surgeons along with general surgery or ENT department,
- quality management of hand surgery along with orthopaedic units,
- quality management of hypospadias/epispadias patients in absence of urosurgeons/paediatric surgeons,
- reducing morbidity in orthopaedic patients by providing early coverage of wounds,
- providing specialized care of burns along with general surgery people.

There is increasing demand for plastic surgery services because of growing public awareness for complete cure, reduction of morbidity, and limitation of deformity which indirectly helps in productivity of population. There is need for realization of problem, setting up priorities to develop infrastructure, laying down protocol, and publicity to educate people [2, 9].

## 7. Conclusion

Marginal and often barely existent plastic surgical care for most people is a formidable problem experienced in many developing countries worldwide. The cause is multifactorial, existing in part because of insufficient financial support, lack of organizational structure, lack of trained local plastic surgeons, coupled with the glamorous image of specialty which apparently makes it nonessential for common man. 

Plastic surgical expertise is necessary for waste majority of patients to provide them optimum cure and hence it has a legitimate role in improving health care status in developing countries.

## Figures and Tables

**Table 1:** Patient distribution.

Total number of patients	848
Acute burn patients	307
Plastic surgery patients	541
Operative	296
Minor out patient procedures	245

**Table 2:** Contribution of different departments.

Departments	No of procedures
General Surgery	263
Orthopedics	20
Otorhinolaryngology	9
Opthalamology	2
Dentistry	2

**Table 3:** Type of procedures.

Type of procedures	*n* = 296
Major (long procedures requiring general/regional anesthsia)	174
	
Intermediate (small procedures requiring general/regional anesthsia)	21
	
Minor (procedures under local anesthesia)	101

**Table 4:** Details of various plastic surgery procedures.

	
Cleft	*n* = 7

Cleft lip repair	4
Palatoplasty	1
cleft lip nose rhinoplasty	2

Oncological reconstructions	*n* = 59

Free radial art forearm flap	3
Free anterolateral thigh flap	1
Free fibula osteocutaneous flap	1
Pec major musculocut flap	7
Deltopectoral flap	2
Facial art musculomucosal flap	1
Abbe estlander flap	2
Scalp rotation flap	2
Scalp transposition flap	2
Latissimus Dorsi Flap	3
TRAM flap	1
Pedicled Anterolateral thigh flap	2
Fasciocutaneous flap lower limb	5
Reverse sural flap	1
Local limberg flaps	15
Skin grafting/direct closure	11

postburn deformities	*n* = 45

Contracture neck	3
Contracture axilla and elbow	8
Contracture wrist	4
Contracture fingers and hand	14
Amputations electric burn	3
Perineal and groin contracture	2
Ectropion	2
Skin Grafting	9

Genitourinary reconstructions	*n* = 12

Hypospadias repair	8
Penile fracture	1
Filarial scrotum and penis	3

Craniofacial and maxillofacial	*n* = 4

Maxillofacial fractures	3
Fracture skull bones with	1
extrusion of brain matter	

Congenital AV malformations	*n* = 20

Haemangioma Excision	6
AV malformation Excision	10
Lymphangioma Excision	4

Hand and upper extremity reconstruction	*n* = 39

Hand reimplantation	3
Multiple finger reimplantation	1
Revascularization hand and forearm	9
Pedicled radial artery forearm flap	5
Free gracilis flap	1
Abdominal flap	5
MP joint capsulotomy	1
Polydactyly	1
Syndactyly	2
Skin grafts, Local flaps,	11

Esthetic Surgery	*n* = 30

Blepharoplasty Asian eyelid	13
Rhinoplasty	1
Gynaecomastia	4
Tatto removal	5
Nevi Excision	7

Lower extremity reconstruction	*n* = 27

Pseudoaneurysm femoral art	4
Vascular repair trauma	2
Gastrocnemius muscle flap	4
Soleus muscle flap	3
Fasciocutaneous transposition flap	8
Anterolateral thigh flap	1
Polydactyly	1
Tendon transfer	1
Dorsalis pedis flap	1
Lat planter rotation flap	2

Pressure sores	*n* = 5

Tensor fascia lata flap	2
Gluteus maximus flap	2
Gracilis musculocutaneous flap	1

Miscellaneous	*n* = 48

Eg Common carotid pseudoaneurysm repair,	
repair of various cysts, sinus, and other conditions	

**Table 5:** Outcome.

Operative plastic surgery patients	Burn patients nonoperated
(i) Mortality–nil	Overall mortality 15.1%
(ii) Significant morbidity: total flap loss-4	(i) Mortality in year 2007 = 17.92%
(a) Free gracilis flap-1	
(b) Ped Anterolateral thigh flap-1	(ii) Mortality in year 2008 = 12.32%
(c) Free ALT flap-1	
(d) Reverse sural flap-1	

(iii) 2-limb salvage surgeries failed, resulted in amputations	
(a) Crush injury leg and knee	
(b) Infected ruptured femoral artery pseudo aneurysm	
